# Dr. Thomas Oliver Keator

**Published:** 1914-10

**Authors:** 


Dr. Thomas Oliver Keator, Dartmouth, 1875, formerly a health officer of Rochester, died at his home in Accord, N. Y., July 6, aged 60.

